# Infections Caused by Carbapenem-Resistant *Enterobacteriaceae*: An Update on Therapeutic Options

**DOI:** 10.3389/fmicb.2019.00080

**Published:** 2019-01-30

**Authors:** Chau-Chyun Sheu, Ya-Ting Chang, Shang-Yi Lin, Yen-Hsu Chen, Po-Ren Hsueh

**Affiliations:** ^1^Division of Pulmonary and Critical Care Medicine, Department of Internal Medicine, Kaohsiung Medical University Hospital, Kaohsiung, Taiwan; ^2^School of Medicine, Sepsis Research Institute, Graduate Institute of Medicine, Graduate Institute of Clinical Medicine, Center of Dengue Fever Control and Research, College of Medicine, Kaohsiung Medical University, Kaohsiung, Taiwan; ^3^Division of Infectious Disease, Department of Internal Medicine, Kaohsiung Medical University Hospital, Kaohsiung, Taiwan; ^4^Department of Internal Medicine, Kaohsiung Municipal Ta-Tung Hospital, Kaohsiung, Taiwan; ^5^Department of Biological Science and Technology, College of Biological Science and Technology, National Chiao Tung University, Hsinchu, Taiwan; ^6^Department of Laboratory Medicine, National Taiwan University Hospital, College of Medicine, National Taiwan University, Taipei, Taiwan; ^7^Department of Internal Medicine, National Taiwan University Hospital, College of Medicine, National Taiwan University, Taipei, Taiwan

**Keywords:** avibactam, carbapenems, carbapenemase, carbapenem-resistant *Enterobacteriaceae*, combination therapy, relebactam, vaborbactam

## Abstract

Carbapenems are considered as last-resort antibiotics for the treatment of infections caused by multidrug-resistant Gram-negative bacteria. With the increasing use of carbapenems in clinical practice, the emergence of carbapenem-resistant pathogens now poses a great threat to human health. Currently, antibiotic options for the treatment of carbapenem-resistant *Enterobacteriaceae* (CRE) are very limited, with polymyxins, tigecycline, fosfomycin, and aminoglycosides as the mainstays of therapy. The need for new and effective anti-CRE therapies is urgent. Here, we describe the current understanding of issues related to CRE and review combination therapeutic strategies for CRE infections, including high-dose tigecycline, high-dose prolonged-infusion of carbapenem, and double carbapenem therapy. We also review the newly available antibiotics which have potential in the future treatment of CRE infections: ceftazidime/avibactam, which is active against KPC and OXA-48 producers; meropenem/vaborbactam, which is active against KPC producers; plazomicin, which is a next-generation aminoglycoside with *in vitro* activity against CRE; and eravacycline, which is a tetracycline class antibacterial with *in vitro* activity against CRE. Although direct evidence for CRE treatment is still lacking and the development of resistance is a concern, these new antibiotics provide additional therapeutic options for CRE infections. Finally, we review other potential anti-CRE antibiotics in development: imipenem/relebactam and cefiderocol. Currently, high-dose and combination strategies that may include the new β-lactam/β-lactamase inhibitors should be considered in severe CRE infections to maximize treatment success. In the future, when more treatment options are available, therapy for CRE infections should be individualized and based on molecular phenotypes of resistance, susceptibility profiles, disease severity, and patient characteristics. More high-quality studies are needed to guide effective treatment for infections caused by CRE.

## Introduction

The increasing prevalence of bacterial resistance to antibiotics is a critical public health problem. Infections caused by antibiotic-resistant bacteria are associated with significant morbidity and mortality worldwide. Many previous efforts to combat multidrug-resistant (MDR) bacteria were focused on methicillin-resistant *Staphylococcus aureus* (MRSA). In recent years, several new therapeutic options for MRSA have become available (David et al., 2017). Currently the major threat of antibiotic-resistant bacteria is from MDR Gram-negative organisms, particularly those which have developed resistance to carbapenem. Along with carbapenem-resistant *Acinetobacter baumannii* (CRAB) and carbapenem-resistant *Pseudomonas aeruginosa* (CRPA), carbapenem-resistant *Enterobacteriaceae* (CRE) are among the top tier of the WHO list of antibiotic-resistant “priority pathogens” that pose the greatest threat to human health (Willyard, 2017).

*Enterobacteriaceae* are common pathogens causing a variety of severe infections, including bloodstream infections (BSIs), community-acquired pneumonia (CAP), hospital-acquired pneumonia (HAP), ventilator-associated pneumonia (VAP), complicated urinary tract infections (cUTIs), and complicated intra-abdominal infections (cIAIs). Therefore, antibiotic resistance in these bacteria has significant clinical and socioeconomic impacts (Lee et al., 2017; Rodriguez-Bano et al., 2018; Ting et al., 2018). As the prevalence of infections caused by extended-spectrum β-lactamase (ESBL)-producing *Enterobacteriaceae* is increasing, the medical community has been forced to use carbapenem as a first-line empirical treatment. The increasing use of carbapenem for possible ESBL infections has led to a more serious problem: the emergence of carbapenemase-producing *Enterobacteriaceae* (CPE) (Sheu et al., 2018).

Carbapenem is a β-lactam antibiotic which inhibits transpeptidases (penicillin-binding proteins) and prevents peptidoglycan synthesis, leading to lytic cell death (Kohanski et al., 2010). The resistance of CRE to carbapenems is generally based on two mechanisms: carbapenemase production or the combination of structural mutations with the production of other β-lactamases, such as AmpC cephalosporinase (AmpC) and ESBL (Tzouvelekis et al., 2012; Munoz-Price et al., 2013; Goodman et al., 2016; Tamma and Simner, 2018). The classification and characteristics of major carbapenemases in CRE are summarized in Table 1. The three major classes of carbapenemases are Ambler Class A *Klebsiella pneumoniae* carbapenemase (KPC); Class B metallo-β-lactamases (MBLs) such as New Delhi MBL (NDM), Verona integrin-encoded MBL (VIM), and imipenemase (IMP); and Class D oxacillinases (OXA)-type enzymes such as OXA-48-like carbapenemases. These carbapenemases exhibit variable levels of carbapenem resistance through their carbapenem-hydrolyzing activity. For instance, a certain proportion of VIM and IMP-producing *K. pneumoniae* have been observed to have low carbapenem minimum inhibitory concentrations (MICs) in studied isolates (Yan et al., 2001; Psichogiou et al., 2008; Daikos et al., 2009). On the other hand, NDM carbapenemase seemed to exhibit higher carbapenem MICs (Kumarasamy et al., 2010) while KPC-producing isolates demonstrated wide variations in carbapenem MICs in different geographic regions (Endimiani et al., 2009a; Daikos and Markogiannakis, 2011; Qi et al., 2011). Some carbapenem-producing *Enterobacteriaceae* (CPE) are even susceptible to carbapenems themselves and this is particularly observed in OXA-48 producers (Dautzenberg et al., 2014; Navarro-San Francisco et al., 2013). Some CPE may also coproduce AmpC or ESBLs. The impact of the co-production of these enzymes on treatment and outcomes remains unclear.

**Table 1:** Classification and characteristics of major carbapenemases in *Enterobacteriaceae*.

Carbapenemase	KPC	MBLs (NDM, VIM, IMP)	OXA-48
Ambler molecular class	A	B	D
Substrates of hydrolysis	All β-lactams	All β-lactams except for aztreonam	Penicillins and carbapenems
Inhibited by classic β-lactamase inhibitors	Minimally	No	No
Inhibited by avibactam	Yes	No	Yes
Inhibited by vaborbactam	Yes	No	No
Inhibited by relebactam	Yes	No	No
Common species in *Enterobacteriaceae*	*K. pneumoniae, E. coli, Enterobacter* spp.	NDM: *K. pneumoniae, E. coli* VIM: *K. pneumoniae* IMP: *K. pneumoniae*	*K. pneumoniae*


*KPC, Klebsiella pneumoniae carbapenemase; MBL, metallo-β-lactamase; NDM, New Delhi metallo-β-lactamase; VIM, Verona integrin-encoded metallo-β-lactamase; IMP, imipenemase; OXA, oxacillinase*

To date, the treatment options for CRE infections remain very limited. Polymyxins (colistin or polymyxin B) and tigecycline have been historically considered as drugs of choice for infections caused by CRE. However, resistance to these antibiotics is increasing (Capone et al., 2013; Giacobbe et al., 2015; Yang et al., 2018). In addition to polymyxins and tigecycline, fosfomycin and aminoglycosides are occasionally used (Tang et al., 2016; Tseng et al., 2017; Rodriguez-Bano et al., 2018). Carbapenems still play a role in the treatment of CRE infections, particularly when used in the treatment of CRE with lower MICs, either in higher doses, in combination with other active anti-CRE agents, or through double-carbapenem therapy (DCT). Older antibiotics such as minocycline, doxycycline, trimethoprim/sulfamethoxazole, and chloramphenicol may be effective for some CRE isolates (Falagas et al., 2011; Livermore et al., 2011). Recently, novel β-lactamase inhibitor combinations have provided new therapeutic options for CRE infections. However, these new β-lactamase inhibitors are not active against all carbapenemases. Avibactam inhibits both Class A KPC and Class D OXA-48 (Zasowski et al., 2015), while vaborbactam and relebactam inhibits only Class A KPC (Petty et al., 2018; Zhanel et al., 2018) (Table 1).

Table 2 summarizes the currently available antimicrobial agents and their recommended doses for treatment of CRE infections. Because the therapeutic options are limited, all potentially active drugs should be tested *in vitro*. It is recommended to select anti-CRE agents according to *in vitro* susceptibility data, clinical severity, and all other available information. Higher doses may be necessary for severe infections, including pneumonia and septic shock (Rodriguez-Bano et al., 2018). Many anti-CRE agents have recently been reviewed (Ni et al., 2016; Thaden et al., 2017; Trecarichi and Tumbarello, 2017; Zavascki et al., 2017; Rodriguez-Bano et al., 2018). We therefore focus the present review on several potential combination therapeutic strategies and new antibiotics (Table 3).

**Table 2:** Antimicrobial agents used for carbapenem-resistant *Enterobacteriaceae* infections.

Antimicrobial agents	Recommended dose for CRE infections^a^	Comments
Meropenem	2 g every 8 h by prolonged infusion for isolates with MICs of 2–8 mg/L	May not be effective for isolates with MIC > 8 mg/L
Ertapenem	Consider 2 g every 24 h	Used in double-carbapenem therapy
Colistin	Loading dose of 9 MU, followed by 9 MU/day in 2–3 divided doses	
Polymyxin B	Loading dose of 2–2.5 mg/kg, followed by 5 mg/kg/day in 2 divided doses	
Tigecycline	Loading dose of 100 mg, followed by 50 mg every 12 h	Consider loading dose of 200 mg, followed by 100 mg every 12 h for severe infections
Eravacycline	1 mg/kg every 12 h	Approved by FDA in August 2018 for the treatment of cIAI. Activity against carbapenem-resistant *Enterobacteriaceae* has been demonstrated *In vitro*. Clinical data in CRE infections are still lacking
Gentamicin Tobramycin	5–7 mg/kg/day	Used in combination therapy. Consider a higher dose of 10–15 mg/kg/day for severe infections without other options. Risk of toxicity may increase. TDM is recommended
Amikacin	15–20 mg/kg/day	Used in combination therapy. Consider a higher dose of 25–30 mg/kg/day for severe infections without other options. Risk of toxicity may increase. TDM is recommended
Plazomicin	15 mg/kg/day	Approved by FDA in June 2018 for the treatment of cUTI including pyelonephritis. Activity against ESBL- and carbapenemase-producing *Enterobacteriaceae* has been demonstrated *In vitro*. Clinical data in CRE infections are still lacking
Fosfomycin	4 g every 6 h to 8 g every 8 h	Used in combination therapy
Aztreonam	1–2 g every 8 h	MBL producers are susceptible if not ESBL or AmpC producers
Ceftazidime	1–2 g every 8 h	OXA-48 producers are susceptible if not ESBL or AmpC producers
Ceftazidime/avibactam	2.5 g (2 g/0.5 g) every 8 h	KPC and OXA-48 producers are frequently susceptible
Meropenem/vaborbactam	2 g (1 g/1 g) every 8 h	KPC producers are frequently susceptible


*cIAI, complicated intraabdominal infection; cUTI, complicated urinary tract infection; ESBL, extended-spectrum β–lactamase; KPC, Klebsiella pneumoniae carbapenemase; MBL, metallo-β-lactamase; MIC, minimum inhibitory concentration; OXA, oxacillinase; TDM, therapeutic drug monitoring.Adapted from Rodriguez-Bano et al. (2018).^*a*^For patients with normal renal function*.

**Table 3:** Potential combination therapeutic strategies and new antibiotics for the treatment of carbapenem-resistant *Enterobacteriaceae* infections.

Combination therapeutic strategies
High-dose tigecycline
High-dose prolonged-infusion of carbapenem
Double-carbapenem therapy
**New antibiotics**
Ceftazidime/avibactam
Meropenem/vaborbactam
Plazomicin
Eravacycline
**New antibiotics in development**
Imipenem/cilastatin and relebactam
Cefiderocol


## Combination Therapeutic Strategies

Many studies have investigated the benefits of various combinations of antimicrobial agents for the treatment of CRE, *in vitro* or *in vivo* (Ku et al., 2017). However, it has been difficult to draw conclusions due to the diversity of study designs and resistance mechanisms. Among the studies that found combination therapy to contribute to lower mortality rates than monotherapy (Qureshi et al., 2012; Tumbarello et al., 2012, 2015; Tofas et al., 2016; Trecarichi et al., 2016; Gutierrez-Gutierrez et al., 2017; Machuca et al., 2017; Papadimitriou-Olivgeris et al., 2017), the two largest retrospective studies to date concordantly identified the protective effects to be significant in populations with high disease severity (Tumbarello et al., 2015; Gutierrez-Gutierrez et al., 2017). In the multicenter Italian cohort, with 661 episodes of BSI and non-BSI caused by KPC-producing *K. pneumoniae*, Tumbarello et al. (2015) compared clinical outcomes between 307 patients receiving monotherapy (colistin in 121, tigecycline in 116, gentamicin in 70) and 354 patients receiving combination therapy (receiving 2 or more *in vitro*-active drugs, with meropenem in all cases). They found significantly decreased mortality rates with combination therapy among patients with BSI (OR, 0.45; 95% CI, 0.29–0.68), lower respiratory tract infections (OR, 0.35; 95% CI, 0.11–0.99), high APACHE III scores (OR, 0.55; 95% CI, 0.37–0.80), septic shock (OR, 0.18; 95% CI, 0.05–0.53), and among isolates with a meropenem MIC of ≤8 mg/L (OR, 0.57; 95% CI, 0.32–1.03) (Tumbarello et al., 2015). The INCREMENT project, which included monomicrobial BSIs due to CPE from a total of 437 patients worldwide (26 tertiary hospitals in 10 countries), is by far the largest retrospective international cohort study. The subgroup analysis concluded that combination therapy, defined as receiving more than one *in vitro*-active antimicrobial, did not improve survival except in patients with a high mortality score (Gutierrez-Gutierrez et al., 2017).

The highly heterogeneous methodologies between studies and the fact that most data were derived from isolates of CRKP, preclude optimal synthesis using the currently available evidence. Several systematic reviews have proposed viewpoints regarding combination therapy. Falagas et al. (2014) reviewed 20 non-randomized studies, comprising 692 patients, and proposed that combination therapy may be considered for severely ill patients. Tzouvelekis et al. (2012) performed a systematic review that included 34 studies and suggested that carbapenem-containing combinations contribute to higher treatment success rates. Polymyxin-based and tigecycline-based combination therapies were reported to significantly decrease 30-day mortality when compared with respective monotherapy by systematic reviews (Ni et al., 2015, 2016). Zusman et al. (2017) performed a meta-analysis to compare polymyxin-based combination therapy and monotherapy. The subgroup analysis for CRE comprised of *K. pneumoniae* BSI and included seven studies with a total of 285 patients. The meta-analysis favored combination therapy (potentially double-coverage) and demonstrated an OR of 2.09 (95% CI, 1.21–3.6; *I*^2^ = 0%), but with low-quality evidence (Zusman et al., 2017). The most recent meta-analysis, performed by Martin et al. (2018), included 22 studies describing CRE infections. Seven studies were extracted for comparison between combination therapy and monotherapy. Four of the studies included patients with BSIs and three with mixed infections. The results showed a significantly higher risk of overall mortality among patients treated with monotherapy (OR, 2.19; 95% CI, 1.00–4.80), with a high heterogeneity (*I*^2^= 84.2%; QP = 0.003) (Martin et al., 2018).

The first and only RCT for the treatment of carbapenem-resistant Gram-negative bacteria was recently published, and is therefore not included in any of the above-mentioned systematic reviews or meta-analyses. The open-label RCT compared the outcomes of colistin monotherapy vs. combination therapy with high-dose and prolonged infusion meropenem (2 g every 8 h, infused over 3 h) (Paul et al., 2018). A total of 406 patients were enrolled, with pneumonia and bacteremia comprising 87% of the infections. Most infections were caused by *A. baumannii* (77%), while *Enterobacteriaceae* only contributed to 18% (73/406) of all infections. Most *Enterobacteriaceae* infections were BSIs (77%), with *K. pneumoniae* being the main pathogen (89%). In the *post hoc* subgroup analysis of *Enterobacteriaceae* infections, there were no significant differences in clinical outcomes between colistin monotherapy and combination therapy with meropenem. However, combination therapy seemed to be associated with a lower clinical failure rate (46% vs. 68%, *P* = 0.185) and a lower 28-day mortality (21% vs. 35%, *P* = 0.235). There is another ongoing RCT (NCT01597973) investigating colistin monotherapy vs. combination with carbapenem in the treatment of bacteremia or pneumonia caused by extensively drug-resistant Gram-negative bacteria. The trial is estimated to be completed in 2021.

Because of the suboptimal quality of the available data, it is not yet possible to make solid recommendations regarding combination therapy in CRE infections. However, there is a growing body of evidence supporting the use of combination therapy, particularly in critically ill patients.

### High-Dose Tigecycline

Besides carbapenems, the most commonly studied high-dose regimen is tigecycline, owing to its non-nephrotoxic nature compared to other potentially active antimicrobial agents for CRE, such as polymyxins and aminoglycosides. A high-dose colistin regimen has been more extensively investigated for its efficacy against carbapenem-resistant *A. baumannii* infections and seldom for CRE (Gibson et al., 2016; Cheng et al., 2018).

A high-dose tigecycline regimen consists of a 200 mg loading dose and a maintenance dose of 100 mg every 12 h, while a standard-dose regimen consists of a loading dose of 100 mg and a maintenance dose of 50 mg every 12 h. One study assessed the efficacy of tigecycline for carbapenem-producing *K. pneumoniae* (CPKP) by using 164 non-duplicate clinical strains of CPKP isolated from HAP and incorporating a Monte Carlo simulation into a pharmacokinetic/pharmacodynamics (PK/PD) model. The study revealed that a higher cumulative fraction of response, indicating better clinical efficacy, can be gained by doubling the tigecycline dose (90.2% vs. 71.2%) (Trecarichi et al., 2016).

Two small retrospective studies conducted by Sbrana et al. (2013) and Balandin Moreno et al. (2014) included 26 episodes of KPC-producing *K. pneumoniae* and 16 episodes of VIM-1-producing *K. pneumoniae* infections from a trauma-referral ICU and a multidisciplinary ICU, respectively. In the study by Sbrana et al. (2013), high-dose tigecycline was administered in 25/26 infection episodes in combination with gentamicin (19/26) and colistin (12/26). Fosfomycin was used as a third antibiotic in 13/26 episodes. In the study by Balandin Moreno et al. (2014), high-dose tigecycline was administered in 10/16 infection episodes and standard-dose regimen in 6/16 episodes. Fourteen (14/16) episodes were treated with combination therapy, including colistin in 8/16, carbapenem in 5/16, ciprofloxacin in 2/16, piperacillin/tazobactam in 1/16, and amikacin in 1/16. Sbrana et al. (2013) suggested a favorable outcome by the double- or triple-combination with high-dose tigecycline, with a 30-day crude mortality rate of 14%. On the other hand, Balandin Moreno et al. (2014) found no significant differences in the mortality rates between high-dose and standard-dose tigecycline. Di Carlo et al. (2013) compared standard-dose to high-dose tigecycline with the combination of colistin in 30 postoperative abdominal surgery ICU patients who had at least two positive blood cultures for KPC-producing *K. pneumoniae*. They observed a significantly lower mortality rate in the high-dose tigecycline group. In terms of infection sources, De Pascale et al. (2014) found high-dose tigecycline to be the only independent predictor of clinical cure in the VAP subgroup of critically ill patients with MDR bacterial infections (total 63 patients, 28 isolates of CRAB and 27 isolates of carbapenem-resistant *K. pneumoniae* [CRKP]). For BSIs, a retrospective cohort study of 40 patients with nosocomial CPKP BSI showed no significant differences in in-hospital mortality between 23 patients undergoing high-dose tigecycline-based combination therapy and 17 patients undergoing standard-dose tigecycline therapy (52.2% vs. 76.5%, *P* = 0.117) (Geng et al., 2018). One systemic review encompassed 25 studies reporting the efficacy and/or safety of tigecycline-based regimens for treating CRE infections, while the subgroup meta-analysis found a much lower ICU mortality with high-dose tigecycline than standard-dose tigecycline (OR, 12.48; 95% CI, 2.06–75.43; *P* = 0.006) (Ni et al., 2016).

In summary, high-dose tigecycline-based combination therapy may be considered in critically ill patients with CRE infections and limited treatment options, either from the PK/PD viewpoint or clinical observations. However, the infection source should be cautiously evaluated since better outcomes were observed in trauma (Sbrana et al., 2013) or postoperative abdominal surgery (Di Carlo et al., 2013) patients, but not in patients with BSI (Geng et al., 2018), which is compatible with the common consensus that tigecycline is extensively distributed beyond the plasma volume and concentrates into tissues.

### High-Dose and Prolonged-Infusion of Carbapenems

The fact that wide disparities of carbapenem MICs exist, even among CPE isolates, complicates the discourse for the role of carbapenems in the treatment of CRE or CPE. Several studies have investigated the efficacy of carbapenems against CPKP in animal models and suggested that with a higher dose of carbapenems it is possible to attain reliable reductions in bacterial density in isolates with lower carbapenem MICs (Daikos et al., 2007; Bulik and Nicolau, 2010b; Bulik et al., 2010a; Souli et al., 2011). Daikos and Markogiannakis (2011) proposed, based on several animal infection model studies, that high-dose, prolonged-infusion carbapenems can achieve bactericidal effects in immunocompetent animals infected by KPC-producing *K. pneumoniae* isolates with MICs up to 8 mg/L. In addition, Daikos and Markogiannakis (2011) also analyzed 22 clinical studies (mostly case series) and found: (1) the therapeutic efficacy of carbapenems increases from 29% for an MIC of >8 mg/L, to 69% for an MIC ≤ 4 mg/L, which is similar to patients infected with non-CPKP (73%); (2) among the 138 patients treated by combination regimens, the mortality rate was lowest in patients who received carbapenem-containing combinations and were infected with isolates of MIC ≤ 4 mg/L (OR, 5.3; 95% CI, 1.5–18.9). More recent retrospective cohort studies echoed the observation that a combination regimen containing carbapenem is associated with significantly higher survival rates in CPKP BSI isolates (Daikos et al., 2014) and KPC-producing *K. pneumoniae* isolates with a meropenem MIC ≤ 8 mg/L (Tumbarello et al., 2015).

In addition to carbapenem-containing combinations, the strategy of high-dose (2 g every 8 h) carbapenem with prolonged infusion (over 3 h) was also found to be associated with better outcomes in CPKP infections (Tumbarello et al., 2012, 2015; Daikos et al., 2014). Moreover, Giannella et al. (2018) evaluated the efficacy of high-dose carbapenem-based combination therapy among 595 patients with CRKP BSI and studied the benefits of high-dose carbapenem in strains with meropenem MIC ≥ 16 mg/L, which comprised 77% of all isolates in the study. These clinical observations are in line with PK/PD studies showing that a high-dose prolonged-infusion carbapenem regimen can reach attainable targets in isolates with meropenem MICs up to 32–64 mg/L, though failure was observed with very high MICs of 256–1024 mg/L (Del Bono et al., 2017; Giannella et al., 2018).

Based on the current evidence, using a high-dose prolonged-infusion carbapenem-containing combination regimen for the treatment of CRE isolates with carbapenem MIC ≤ 8 mg/L may be considered when there are no other treatment options. It should also be noted that since most conclusions from the above-mentioned studies were derived from CPKP or CRKP isolates, the extrapolation of high-dose prolonged-infusion of carbapenem-containing combination therapy to other CRE with different resistance mechanisms requires further investigation.

### Double-Carbapenem Therapy

The most well-investigated DCT is the combination of ertapenem (with a standard infusion time of 30–60 min) prior to a prolonged infusion of meropenem or doripenem over 3–4 h, with high-dose meropenem of 2 g every 8 h being most commonly applied. This regimen originated from the revolutionary approach proposed by Bulik and Nicolau (2011), as a salvage option for CPKP. The study validated enhanced activities for both ertapenem and doripenem in combination, using an *in vitro* chemostat and *in vivo* murine thigh infection model (Bulik and Nicolau, 2011). The rationale for this combination came from the hypothesis that ertapenem might play a sacrificial role, being preferentially hydrolyzed due to its greater affinity to KPC (Anderson et al., 2007), permitting the concomitant administration of carbapenem to sustain a high concentration. Some *in vitro* studies show a beneficial effect of lower MICs of meropenem (MIC ≤ 128 mg/L) (Oliva et al., 2017b) or doripenem (MIC ≤ 16 mg/L) (Wiskirchen et al., 2013) with regard to ertapenem-based DCT. Nevertheless, ertapenem-based DCT is not the only combination that demonstrates synergism (Poirel et al., 2016; Fredborg et al., 2017). One *in vitro* study reported variable synergistic patterns among KPC and OXA-48 producers, while no synergism was observed for the NDM-producing strain (Poirel et al., 2016).

Despite the diversity of the above-mentioned observations, nearly all reported cases and clinical studies adopted an ertapenem-based DCT with recommended or high-dose doripenem/meropenem. The short stability of the intravenous imipenem preparation that hinders prolonged infusion may be one of the reasons for such a phenomenon (Mashni et al., 2018). Souli et al. (2017) conducted the largest observational cohort study to date in terms of exclusive DCT as a salvage therapy. The study included 27 patients with CPKP infections, mostly with cUTI (59.3%) and BSI (48.2%) and reported a high clinical success rate of 77.8%. It is noteworthy that the subgroup of pandrug-resistant infections also had a successful clinical and microbiological outcome of 78.5% (11/14). Among critically ill patients with severe sepsis or septic shock, a successful outcome was noted in 81.8% (9/11) (Souli et al., 2017). Oliva et al. (2017a) and Venugopalan et al. (2017), both conducted observational comparator studies to compare the efficacy of DCT. Venugopalan et al. (2017) enrolled 36 patients with CRKP bacteremia, including 18 patients receiving doripenem and ertapenem (DCT group), and 18 patients receiving doripenem and colistin (control group). They found the DCT group had a significantly improved clinical cure rate of 72% (13/18, vs. control group of 39%, 7/18; *P* = 0.049) and a lower 30-day mortality of 31%, with a trend toward statistical significance (vs. 61%, *P* = 0.087) (Venugopalan et al., 2017). Oliva et al. (2017a) enrolled 32 patients with CRKP infections, including 18 patients receiving ertapenem and meropenem, and 14 patients receiving ertapenem, meropenem, and colistin. They found the combination of colistin and DCT obtained rapid bactericidal activity up to 24 h in the *in vitro* analysis. However, there were no significant differences regarding the early response or 60-day mortality between the two groups. Therefore, the author concluded that the addition of colistin to DCT should be considered in severe cases with septic shock at presentation, then withdrawn after clinical stabilization with a stable switch to the less nephrotoxic regimen of DCT (Oliva et al., 2017a). A randomized controlled trial (RCT) addressing this topic has not yet been performed. The only matched case-control study to date included 48 patients with a DCT-containing combination (daily dose of meropenem and ertapenem up to 6 and 2 g, respectively), matched with 96 controls of DCT-sparing regimens. The other concomitant antibiotics comprised colistin (9 MU every 12 h), gentamicin (5–7 mg/kg daily) and high dose tigecycline. The 28-day mortality was significantly higher in the DCT-sparing arm (47.9% vs. 29.2%, *P* = 0.04). In multivariate analysis, the DCT-containing regimen was associated with a reduction in 28-day mortality (OR, 0.43; 95% CI, 0.23–0.79) (De Pascale et al., 2017). Mashni et al. (2018) performed a critical review of the current studies investigating DCT for CPKP infections, which contained eight case reports and six clinical studies (a total of 171 patients). Most patients were critically ill, and all were treated with ertapenem followed by a prolonged infusion of meropenem or doripenem. Clinical and microbiological successes were reported in approximately 70% of the patients and mortality in 24%. Adverse events, most frequently seizures, sodium disorders, and gastrointestinal symptoms, were reported in 16 patients, without the requirement for treatment interruption (Mashni et al., 2018).

Double-carbapenem therapy seems promising based on current reports, though the majority of infections involved in the studies were caused by Class A carbapenemase or KPC-producing *K. pneumoniae*. The efficacy of DCT against MBLs, such as NDM, would require further investigation.

## New Antibiotics

### Ceftazidime/Avibactam

Ceftazidime/avibactam (CAZ/AVI, AvyCaz^®^, Allergan Inc., Jersey City, NJ, United States) is a new β-lactam/β-lactamase inhibitor combination recently approved for the treatment of cIAIs and cUTIs in the United States in February 2015 (Kaye and Pogue, 2015), and for the treatment of HAP and VAP in January 2018. Unlike most β-lactamase inhibitors, avibactam is not a β-lactam. Avibactam is a novel synthetic non-β-lactam (diazabicyclooctane)/β-lactamase inhibitor that inhibits a wide range of β-lactamases, including Ambler Class A (GEM, SHV, CTX-M, and KPC), Class C (AmpC), and some Class D (OXA-48) β-lactamases (de Jonge et al., 2016). It does not inhibit Class B MBLs (IMP, VIM, VEB, and NDM) (Syue et al., 2016; Wong and van Duin, 2017). The addition of avibactam restores ceftazidime activity against various *Enterobacteriaceae* and *P. aeruginosa*, therefore expanding the activity spectrum of ceftazidime to MDR Gram-negative bacteria.

*In vitro* studies of CAZ/AVI showed adequate efficiency against CRE isolates (Castanheira et al., 2015; Dupont et al., 2016). However, clinical data on the efficacy of CAZ/AVI in severe infections caused by CRE are still lacking. At the time of writing this review, only one prospective, multicenter, observational study (van Duin et al., 2018) and a few case series and cohort studies describing the use of CAZ/AVI for treating CRE infections had been published (Shields et al., 2016, 2017b; Castón et al., 2017; King et al., 2017; Krapp et al., 2017; Temkin et al., 2017). Three studies have compared treatment with CAZ/AVI and other agents for CRE infections (Table 4) (Castón et al., 2017; Shields et al., 2017b; van Duin et al., 2018). The first study enrolled hematologic patients with CRE bacteremia in Spain and Israel (Castón et al., 2017). Compared to patients treated with other agents (*n* = 23), the patients receiving CAZ/AVI (*n* = 8) had a higher 14-day clinical cure rate in univariate analysis (85.7% [6/8] vs. 34.8% [8/23], *P* = 0.031). However, there was a lack of statistical significance in multivariate analysis due to the small number of cases. Another study of patients with CRE bacteremia showed that 13 patients treated with CAZ/AVI had a higher clinical success rates, compared to 96 patients treated with other regimens (85% [11/13] vs. 40.6% [39/96], *P* = 0.003) (Shields et al., 2017b). Although a better clinical response was consistent in multivariate analysis, these results were limited by small case numbers with CAZ/AVI treatment and a potential bias in the selection of therapy. Finally, the first prospective cohort study to compare the clinical outcomes for patients with CRE infections was recently published (van Duin et al., 2018). This observational study compared 38 patients treated with CAZ/AVI to 99 patients treated with colistin for KPC-producing CRE infections; combination therapy was used in 63 and 94% of patients treated with CAZ/AVI and colistin, respectively. Primary BSI was the most common infection foci (46%), followed by HAP (22%). Adjusted all-cause mortality was significantly lower in the CAZ/AVI group (absolute difference 23%; 95% CI, 9% to 35%; *P* = 0.001). A multicenter, retrospective study reviewed 60 patients receiving CAZ/AVI for CRE infections (King et al., 2017). The authors reported an overall in-hospital mortality rate of 32%, a microbiological cure rate of 53%, and a clinical success rate of 65%. There was no significant difference in the in-hospital mortality rate between patients receiving CAZ/AVI monotherapy vs. CAZ/AVI combination therapy (30% [10/33] vs. 33% [9/27], *P* = 1.0), and between patients with bacteremia vs. those without (39% [9/23] vs. 27% [10/37], *P* = 0.397). Shields et al. (2016) reported a case series in a single center including 37 cases of patients with CRE infections who received treatment with CAZ/AVI. The survival rate at 30 days was 76% (28/37). Clinical success was observed as 59% (22/37), with no significant difference between patients receiving monotherapy vs. combination therapy (58% [15/26] vs. 64% [7/11]). However, they also reported a CRE infection recurrence rate of 23% (5/22) among patients who had displayed clinical success, and an overall microbiologic failure rate of 27% (10/37). Temkin et al. (2017) reported a case series of 38 patients with CRE (*n* = 36) and *P. aeruginosa* (*n* = 2) infections treated with CAZ/AVI. Of these, 65.8% (25/38) of patients concurrently received other regimens. The overall clinical and/or microbiological cure rate was 73.7% (28/38), with 69.2% (9/13) in the monotherapy group and 76.0% (19/25) in the combination therapy group.

**Table 4:** Efficacy of ceftazidime/avibactam for treatment of carbapenem-resistant *Enterobacteriaceae* infections.

Reference	Study place, year	Study design	Patients, number	Infection foci	Organism(s)/β-lactamase types	Antibiotic(s)	Clinical outcomes	Remarks
van Duin et al., 2018	United States, 8 sites, 2011–2015	Prospective	137	BSI (*n* = 63; 46%) and pneumonia (*n* = 30, 22%)	97% (133/137) were *K. pneumoniae*	CAZ/AVI (*n* = 38) vs. colistin (*n* = 99)	Adjusted all-cause mortality was significantly lower in the CAZ/AVI group (9% vs. 32%, *P* = 0.001)	Prospective, observational study on the use of CAZ/AVI compared to colistin specifically for infections due to CRE, including 30 (22%) patients with pneumonia
Castón et al., 2017	Spain, Israel, multicenter, 2012–2016	Retrospective	31, all with hematologic malignancy	Primary BSI (*n* = 14, 45.2%)	80.6% (25/31) were *K. pneumoniae*	CAZ/AVI (*n* = 8) vs. others (*n* = 23)	14-day clinical cure rate was higher in the CAZ/AVI group (85.7% [6/8] vs. 34.8% [8/23], *P* = 0.031)	Small case numbers; no difference in crude mortality
Shields et al., 2017b	United States, single site, 2009-2017	Retrospective	109	Secondary bacteremia resulted from abdominal (46%, 50/109)	97% (106/109) of *K. pneumoniae* harbored *bla*_KPC_	CAZ/AVI (*n* = 13) vs. others (*n* = 96)	CAZ/AVI group had higher clinical success rates (85% [11/13] vs. 40.6% [39/96], *P* = 0.003)	Small case numbers with CAZ/AVI treatment; bias in selection of therapy


*CAZ/AVI, ceftazidime/avibactam; BSI, bloodstream infection*.

As described earlier, avibactam inhibits Ambler Class A (KPC) and Class D (OXA-48) but did not inhibit Class B MBLs (NDM, VIM, and IMP). Therefore, CAZ/AVI is not active against all CRE isolates (Falcone et al., 2018). In contrast, the monobactam antibiotic aztreonam (ATM) is stable against MBLs but is hydrolyzed by many other β-lactamases (ESBL, AmpC, and cephalosporinases) frequently co-produced by the MBL-producing strains (Marshall et al., 2017). The combination of CAZ/AVI and ATM has been proposed as a potential therapeutic strategy against infections caused by MBL-producing bacteria. Crandon and Nicolau (2013) tested the efficacy of CAZ/AVI and ATM using the neutropenic-mouse thigh infection model, and concluded that this combination represents an attractive treatment option for infections caused by MBL-producing strains that co-produce ESBLs or AmpC. Although studies have demonstrated good *in vitro* activity of CAZ/AVI and ATM against MBL-producing *Enterobacteriaceae* (Marshall et al., 2017; Wenzler et al., 2017), clinical data are still lacking.

These observational studies are subject to selection bias. Further RCTs are warranted to evaluate the effectiveness of CAZ/AVI, as well as CAZ/AVI and ATM combination, for the treatment of CRE infections. Whether CAZ/AVI combination therapy is more effective than monotherapy for deep-seat infections or high-risk patients also needs further evaluation. Moreover, the emergence of CAZ/AVI resistant strains during treatment has been reported. Shields et al. detected CAZ/AVI resistance due to mutations in the *bla*_rmKPC-3_ gene in 3 of 10 microbiologic failures, following CAZ/AVI treatment for 10–19 days (Shields et al., 2016, 2017a). Clinicians should be aware of the possible emergence of resistance following treatment with CAZ/AVI.

### Meropenem/Vaborbactam

Vaborbactam is a novel boron-containing serine-β lactamase inhibitor which confers activity against certain meropenem-resistant bacteria by inhibiting Ambler Class A and C serine carbapenemases, such as KPC (Castanheira et al., 2017). However, it has no *in vitro* activity against Class B metallo-β-lactamases producers (NDM or VIM) or Class D OXA-48 β-lactamases (Castanheira et al., 2016; Nelson et al., 2017). Meropenem/vaborbactam (MER/VAB) (Vabomere^®^, The Medicines Company, Parsippany, NJ, United States) is a novel carbapenem/β-lactamase inhibitor antimicrobial agent approved in August 2017 by the United States Food and Drug Administration (FDA) for the treatment of cUTIs, including pyelonephritis.

MER/VAB exhibited effective *in vitro* antibacterial activity against CRE isolates (Castanheira et al., 2017; Pfaller et al., 2018), with susceptibility rates ranging from 66.2 to 100% (Dhillon, 2018). The clinical data supporting MER/VAB for CRE infections is from the multicenter, randomized, open-label Tango II trial comparing the efficacy and safety of MER/VAB with the best available therapy (BAT) for the treatment of serious CRE infections (Wunderink et al., 2018). Of the 77 enrolled patients, 47 had confirmed CRE infections, including 22 BSI, 16 cUTI/pyelonephritis, 5 HAP/VAP, and 4 cIAI. The results showed statistical significance in favor of MER/VAB over BAT for a clinical cure (65.6% [21/32] vs. 33.3% [5/15], *P* = 0.03) and 28-day mortality (15.6% [5/32] vs. 33.3% [5/15], *P* = 0.03). Furthermore, MER/VAB was associated with decreased nephrotoxicity as compared with BAT (4.0% vs. 24%) (Wunderink et al., 2018). Data for MER/VAB on CRE infections are still accumulating. A study of MER/VAB vs. piperacillin/tazobactam on HAP/VAP (TANGO III, NCT02168946) is ongoing.

### Plazomicin

Plazomicin is a next-generation aminoglycoside synthetically derived from sisomicin, which retains activity against bacteria containing aminoglycoside-modifying enzymes (Landman et al., 2010; Castanheira et al., 2018a). Plazomicin (Zemdri^TM^, Achaogen, Inc., San Francisco, CA, United States) was approved in June 2018 by the FDA for the treatment of adults with cUTI including pyelonephritis who have limited or no alternative treatment options, with a recommended dose of 15 mg/kg every 24 h for normal renal function. The approval was based on two phase 3 clinical trials comparing the efficacy and safety of plazomicin with meropenem (NCT02486627, not published yet) and levofloxacin (NCT01096849, not published yet) for the treatment of cUTI and acute pyelonephritis. Plazomicin demonstrates a broad-spectrum activity against Gram-positive cocci and Gram-negative bacilli, including ESBL producers and CRE (Walkty et al., 2014; Karaiskos et al., 2015). Studies have shown that plazomicin is more potent than other aminoglycosides against KPC-producing *Enterobacteriaceae* (Endimiani et al., 2009b). A recent study evaluating the activity of plazomicin and comparators against clinical isolates showed that isolates carried 16S rRNA methyltransferases were resistant to all available aminoglycosides and had elevated plazomicin MICs (Castanheira et al., 2018b). The methyltransferase enzymes commonly found in MBL producers still render bacteria resistant to plazomicin.

The clinical data supporting plazomicin for the treatment of serious infections due to CRE is from a multicenter, randomized, open-label study comparing the efficacy and safety of plazomicin vs. colistin (both in combination with tigecycline or meropenem) (CARE trial, NCT01970371). Although the full report of this trial is not yet available, preliminary analysis shows that the plazomicin group had a significantly lower 28-day mortality compared to the colistin group (7.1% [1/14] vs. 40.0% [6/15]; difference, -32.9%; 95% CI, -60.1% to -4.0%) (McKinnell et al., 2017). In addition to a lower mortality rate, it was well-tolerated and was associated with a lower incidence of serum creatinine elevations. However, these data should be interpreted with caution due to the small sample size.

### Eravacycline

Eravacycline, a synthetic fluorocycline antibacterial agent of the tetracycline class, has broad-spectrum antimicrobial activity against Gram-positive, Gram-negative, and anaerobic bacteria, exception for *P. aeruginosa* (Zhanel et al., 2016). Eravacycline (Xerava^TM^, Tetraphase Pharmaceuticals, Inc., Watertown, MA, United States) was approved by the FDA in August 2018 for the treatment of cIAIs, based on two phase 3 clinical trials which demonstrated statistical non-inferiority of eravacycline to two commonly used comparators: ertapenem (IGNITE1 trial) (Solomkin et al., 2017) and meropenem (IGNITE4 trial, full data not published yet). The recommended dosage is 1 mg/kg intravenous infusion every 12 h. Of note, eravacycline is not indicated for the treatment of cUTI because clinical trials (NCT01978938 and NCT03032510) did not demonstrate the efficacy for the combined endpoints of clinical cure and microbiological success. Eravacycline has been shown to have effective *in vitro* activity against MDR pathogens (Sutcliffe et al., 2013; Livermore et al., 2016; Zhang et al., 2016), with twofold higher activity than tigecycline against CRE (Livermore et al., 2016). However, there is limited clinical data for the treatment efficacy of eravacycline in CRE infections.

## Potential Antibiotics in Development

### Imipenem/Cilastatin and Relebactam

Imipenem/cilastatin and relebactam (IMI/REL, Merck & Co. Inc., Kenilworth, NJ, United States) combines an approved carbapenem with a novel β-lactamase inhibitor. The chemical structure of relebactam is similar to avibactam (Watkins et al., 2013). Like avibactam, relebactam contains a diazabicyclooctane core, which covalently and reversibly binds Class A and C β-lactamases *in vitro*, with an inhibitory mechanism similar to that of avibactam (Blizzard et al., 2014). However, relebactam cannot inhibit Class D OXA-48 like avibactam (Petty et al., 2018; Zhanel et al., 2018). Relebactam potentiates imipenem activity against imipenem-non-susceptible *Enterobacteriaceae* (Karlowsky et al., 2018). The clinical efficacy and safety of IMI/REL has been shown in the phase 2 studies for cUTIs (NCT01505634) (Sims et al., 2017) and cIAIs (NCT01506271) (Lucasti et al., 2016).

The RESTORE-IMI 1 study (NCT02452047) is a multicenter, randomized, double-blind, comparator-controlled trial, comparing the efficacy and safety of IMI/REL vs. colistin plus imipenem/cilastatin (COL + IMI) in patients with imipenem-non-susceptible bacterial infections. Patients with HAP/VAP, cIAI, or cUTI due to imipenem-non-susceptible pathogens, were randomized 2:1 to receive IMI/REL or COL + IMI. In this study, 31 of 47 randomized and treated patients met mMITT criteria. Favorable clinical responses at Day 28 were comparable in the IMI/REL and the COL + IMI arms (71.4% [15/21] vs. 70.0% [7/10]), and the 28-day all-cause mortality was lower in the IMI/REL arm compared to the COL + IMI arm (9.5% [2/21] vs. 30.0% [3/10]) (presented by Motsch et al. at the European Congress of Clinical Microbiology and Infectious Diseases; April 22, 2018; Madrid, Spain). In addition, treatment-emergent nephrotoxicity was lower with IMI/REL compared to COL + IMI (10.3% [3/29] vs. 56.3% [9/16], *P* = 0.002) (presented by Brown et al. at IDWeek, October 6, 2018; San Francisco, CA, United States).

### Cefiderocol

Cefiderocol is the first siderophore antibiotic to advance into late-stage development. Cefiderocol, a novel siderophore cephalosporin, exhibits potent *in vitro* and *in vivo* activity against a variety of Gram-negative bacteria, including CRE (Saisho et al., 2018). Cefiderocol has a unique antibacterial mechanism in which its catechol side chain of binds to ferric acid, with the complex then being actively transported into bacteria via bacterial iron transporters (Ito et al., 2016). In addition, cefiderocol is also highly active against carbapenemase hydrolysis (Wright et al., 2017). Cefiderocol has demonstrated potent *in vitro* activity against CRE isolates, with 97.0% (991/1,022) of isolates demonstrating cefiderocol MICs of ≤4 mg/L (Hackel et al., 2018). A multicenter, randomized, open-label CREDIBLE-CR trial is ongoing to assess the efficacy of cefiderocol, compared to BAT, for severe infections caused by carbapenem-resistant Gram-negative pathogens (NCT02714595).

## Conclusion

The increasing prevalence of CRE infections represents a major threat to human health. Effective antibiotics against CRE remain very limited, with polymyxins, tigecycline, fosfomycin, and aminoglycoside being the mainstays of anti-CRE therapy. With the high mortality of CRE infections and increasing resistance to available antibiotics, it is urgent for the medical community to develop new and effective therapeutic strategies. The first potential strategy is to increase the doses of anti-CRE agents. High-dose carbapenem, colistin, and tigecycline have been associated with better clinical outcomes in CRE infections. The second potential strategy is to combine these anti-CRE agents. Although large-scale observational studies and systematic reviews suggest that combination therapy may be beneficial to patients with severe CRE infections, the quality of evidence is low due to substantial heterogeneity in study design and patient population. A recent RCT showed no benefit of colistin combination therapy with meropenem when compared to colistin monotherapy. The debate on whether or not these agents should be used in combination will continue. DCT with ertapenem infusion prior to a high-dose meropenem or doripenem infusion has been adopted as a salvage therapy for critically ill patients with CRE infections. Studies suggest that a combination of colistin or tigecycline with DCT might be a reasonable strategy for severe CRE infections. However, the target patients and molecular phenotypes of CRE that would benefit from DCT require further investigation. The recent introduction of the novel β-lactamase inhibitors avibactam, vaborbactam, and relebactam, has provided additional therapeutic options for CRE infections. Of note, these new β-lactamase inhibitors are not active against all major carbapenemases. Avibactam inhibits KPC and OXA-48, while vaborbactam and relebactam inhibit only KPC. Expectantly, these novel β-lactamase inhibitor combinations will also provide carbapenem-sparing options for the treatment of MDR bacteria and help control the rapid emergence of carbapenemase-producing bacteria. The next-generation aminoglycoside plazomicin and the fully synthetic tetracycline antibiotic eravacycline, both recently approved by the FDA, may provide us alternative therapeutic options for CRE infections. The activity of plazomicin and eravacycline against CPE has been demonstrated *in vitro*. However, we need more clinical data to support their use in the treatment of serious infections due to CRE. Many other new antibiotics with potential anti-CRE activity are in various stages of development, including the novel β-lactamase inhibitor combination IMI/REL and the novel siderophore cephalosporin cefiderocol. Currently, high-dose and combination strategies that may include the new β-lactam/β-lactamase inhibitors should be considered for severe CRE infections to maximize treatment success.

## Perspective

In the future when more treatment options are available, anti-CRE therapy should be individualized and based on molecular phenotypes of resistance, susceptibility profiles, disease severity, and patient characteristics. More clinical trials are needed to provide high-quality evidence and guide the selection of effective anti-CRE strategies.

## Author Contributions

C-CS, Y-TC, and S-YL collected and analyzed the data, and wrote the manuscript. Y-HC and P-RH revised and edited the manuscript. C-CS, Y-TC, S-YL, Y-HC, and P-RH read and approved the final version of the manuscript.

## Conflict of Interest Statement

The authors declare that the research was conducted in the absence of any commercial or financial relationships that could be construed as a potential conflict of interest.
